# The Challenges of Electronic Health Records and Diabetes Electronic Prescribing: Implications for Safety Net Care for Diverse Populations

**DOI:** 10.1155/2017/8983237

**Published:** 2017-01-18

**Authors:** Neda Ratanawongsa, Lenny L. S. Chan, Michelle M. Fouts, Elizabeth J. Murphy

**Affiliations:** ^1^Division of General Internal Medicine, Department of Medicine, UCSF Center for Vulnerable Populations, University of California, San Francisco, 1001 Potrero Avenue, Box 1364, San Francisco, CA 94143, USA; ^2^San Francisco Department of Public Health, 1001 Potrero Avenue, San Francisco, CA 94110, USA; ^3^Laguna Honda Hospital and Rehabilitation Center, 375 Laguna Honda Blvd, San Francisco, CA 94116, USA; ^4^Division of Endocrinology, Department of Medicine, University of California, San Francisco, 1001 Potrero Avenue, Box 0862, San Francisco, CA 94143, USA

## Abstract

Widespread electronic health record (EHR) implementation creates new challenges in the diabetes care of complex and diverse populations, including safe medication prescribing for patients with limited health literacy and limited English proficiency. This review highlights how the EHR electronic prescribing transformation has affected diabetes care for vulnerable patients and offers recommendations for improving patient safety through EHR electronic prescribing design, implementation, policy, and research. Specifically, we present evidence for (1) the adoption of RxNorm; (2) standardized naming and picklist options for high alert medications such as insulin; (3) the widespread implementation of universal medication schedule and language-concordant labels, with the expansion of electronic prescription 140-character limit; (4) enhanced bidirectional communication with pharmacy partners; and (5) informatics and implementation research in safety net healthcare systems to examine how EHR tools and practices affect diverse vulnerable populations.

## 1. Introduction

*Mrs. D, a 67-year-old Latina woman, suffers from thirst and frequent urination, with a hemoglobin A1c that jumped from 7.5% to 9.7%. Dr. P notes in the pharmacy claims section of the electronic health record (EHR) that Mrs. D has not filled her pioglitazone in 6 months. Mrs. D reluctantly admits she did not like the medication because her face and legs felt swollen. So, Dr. P counseled Mrs. D about adding a short-acting mealtime insulin to her metformin and glargine regimen. Using the EHR electronic prescribing feature, Dr. P tried to submit a prescription to her local pharmacy for Humulin R insulin but was warned by the formulary check that this was not covered. So, Dr. P prescribed Novolin R insulin, typing into the “Take” field “5 units with breakfast and dinner” while leaving the defaulted frequency as “twice daily.”*

*One week later, Mrs. D returns to Dr. P after three episodes of feeling “shaky” and “sweaty” prompted her to stop this new insulin. She shows Dr. P an insulin vial with Novolog, a fast-acting aspart insulin, rather than the regular insulin Novolin R insulin. In addition, Mrs. D has been taking her new insulin when she wakes up and at bedtime because the bottle said “twice daily.” Finally, Mrs. D showed a newly dispensed bottle of pioglitazone and said she was confused about whether she was supposed to restart that medication.*

Rapid deployment of electronic health record (EHR) systems across US safety net clinics has transformed the care delivery system for vulnerable patients. Fueled by 2009 Health Information Technology for Economic and Clinical Health Act, the percentage of outpatient clinics with any type of EHR doubled from 42% in 2008 to 83% in 2014 [1]. Meaningful use requires clinicians to document and place orders for medical care in structured and reportable ways, including the electronic recording and prescribing of medications [2–4]. Federally qualified health centers (FQHC)—previously lacking resources and support to implement EHRs—are now participating in this transformation, with high rates of adoption [5].

EHRs are a necessary and valuable tool for healthcare delivery, with extensive research investigating EHRs' impact on the quality of care delivery, including medication safety [4, 6–11]. However, studies have also raised concerns that the design and implementation of EHR computerized provider order entry (CPOE) may either fail to mitigate or introduce new medication errors [12–17]. This may pose particular risks in safety net clinics, which serve a disproportionately high number of patients with limited health literacy (LHL) and limited English proficiency (LEP), who are shown to experience disparities in communication and care [18–27].

In this article, we highlight how EHR electronic prescribing has affected diabetes care for vulnerable patients and suggest recommendations for EHR design, implementation, policy, and research. Specifically, we present evidence for (1) the adoption of RxNorm; (2) standardized naming and picklist options for high alert medications such as insulin; (3) the widespread implementation of universal medication schedule and language-concordant labels, with the expansion of electronic prescription 140-character limit; (4) enhanced bidirectional communication with pharmacy partners; and (5) informatics and implementation research in safety net healthcare systems to examine how EHR tools and practices affect diverse vulnerable populations.

## 2. Potential EHR Benefits in Medication Safety and Adherence for Outpatient Diabetes Care

### 2.1. Adverse Drug Events

In the US, approximately 4.5 million ambulatory visits relate to adverse drug events (ADE) each year, with the majority of these occurring in outpatient office practices [28]. Patient with diabetes may be at particular risk, with cardiovascular and hypoglycemic medications comprising the majority of preventable ADE [29].

Two decades ago, computerized prescribing was heralded as a potentially powerful tool to improve medication safety and promote more evidence-based prescribing in ambulatory medical care [30, 31]. However, studies have raised concerns about the potential errors resulting from electronic prescribing platforms in ambulatory care. Research shows electronic prescription error rates ranging from 5 to 38% [16], which is similar to rates reported in the era before computerized prescribing [32]. In addition, studies have shown that 16–19% of electronic prescriptions convey contradictory information within a single prescription, with 2.7% of all prescriptions having the potential for a severe ADE [15, 33]. Meanwhile, although electronic prescribing platforms technically support the ability to discontinue medications, these electronic messages incur charges to pharmacies and thus may not be accepted, leaving providers to embed discontinuation instructions in free-text fields [33–35]. Patients with diabetes may be disproportionately affected, as a study showed that, among the top nine medications dispensed after EHR discontinuation, seven were commonly used in diabetes, hypertension, and hyperlipidemia [36]. Overall, whether EHR platforms mitigate error may depend on the specific implementation within individual institutions, as illustrated by a study showing that the same medication-related decision support knowledge base was associated with ADE decreases in one major academic medical center and increases in another [37].

### 2.2. Patient-Centered Prescribing

Two features of EHRs offer the potential to enhance the patient-centeredness of treatment decision-making: formulary support and pharmacy fill data. Cost remains a major and modifiable barrier to medication adherence, particularly for low-income patients [38, 39]. By integrating formulary checks into the clinical decision support of electronic prescribing, EHRs can make cost and formulary information readily available to the prescribing provider. This could reduce delays in patients receiving their medications and reduce patient's out-of-pocket costs thereby improving adherence [11]. In addition, EHRs may also permit clinicians to view claims data from pharmacies, providing a surrogate measure of medication adherence and information about what medications are dispensed outside of the prescribers' healthcare system [40–42]. Both of these tools can facilitate patient-centered discussions about patient's beliefs, concerns, and behaviors around medications, promoting more treatment decision-making tailored to the individual patient and enhancing future medication adherence.

Despite the promise of these electronic prescribing features, it is not clear how much these tools lead to more patient-centered prescribing. For example, a study of interruptive formulary decision support (in which EHRs stop prescribers from moving forward) shifted prescriptions towards preferred tier medications, but patients' out-of-pocket costs were only slightly lower ($10.60 versus $11.81 for angiotensin receptor blockers) and adherence rates did not improve [43].

## 3. Specific Limitations and Recommendations for Current EHRs in Caring for Vulnerable Patients with Diabetes

EHRs and electronic prescribing provide opportunities for improved safety, but they also provide opportunities for new types of errors.

### 3.1. Requiring Brand Name Prescriptions

Both providers and patients are at risk for confusion by databases that force the use of specific brand names based on the National Drug Code (NDC) Directory, which identifies each medication uniquely based on its manufacturer, product formulation, and package size [44]. By forcing the prescriber to overspecify the medication choice, electronic prescribing platforms remove the pharmacist's ability to select a medication that meets the prescriber's true intentions, taking into account available formulations, patients' prescription drug history, and cost and formulary considerations [12, 17]. For example, when EHRs force providers to prescribe regular insulin by selecting a brand name, pharmacists cannot offer patients the formulary or lower cost brand without contacting the provider for a change. This can lead to delays in dispensing or increased cost-related medication nonadherence in low-income patients.

In the case above, Dr. P selected Novolog from the EHR screen in a hurry when she meant to prescribe Novolin R. She likely would have realized her mistake if she was presented with both the brand and generic names.

Thus, some have recommended using RxNorm, a list using standard terminology that contains all medications available on US market that is maintained by National Library of Medicine, as the standardized identifier for choosing and prescribing medications [12]. This labeling would also comply with Joint Commission best practices of including both the generic and the brand name of a medication on the label and EHR, to minimize confusion and error when switching formulations is required [45, 46].

### 3.2. Errors Associated with Insulin Electronic Prescribing

The Institute for Safe Medication Practices classifies insulin as a high alert medication, “drugs that bear a heightened risk of causing significant patient harm when used in error” [47]. EHRs pose specific risks around insulin prescribing and medication reconciliation.

EHR medication searches may list similar medication names in close proximity on the screen, which increases the risk of selecting the wrong medication [48–50] This is particularly problematic for insulin, since many insulin products have similar brand or generic names but differ in onset and duration of action [51]. The use of “tall man letters,” for example, distinguishing “NovoLOG” versus “NovoLIN,” can reduce such risk [52]. In addition, insulin formulations may be up to 5 times more concentrated than the standard U-100 (100 units per mL) insulin: for example, U-200 (200 units per mL) insulin lispro, U-300 (300 units per mL) insulin glargine, and U-500 (500 units per mL) regular insulin. Although providers may not know that this concentrated insulin exists, EHR databases may display these selections side by side with similarly named standard formulations, introducing new risks of confusion and ADE.

Moreover, the vendor-approved terminology for a particular medication may be unfamiliar to prescribing providers. For example, a standardized e-Prescribing terminology for NPH insulin, “insulin isophane,” is not used in educational or clinical literature, and after unsuccessfully searching for “NPH,” providers may erroneously select an insulin formulation with a different onset or concentration.

Provider training is not sufficient to prevent these kinds of errors; thus software vendors should commit to adopting safe medication practices for high alert medications like insulin.

### 3.3. Sig Confusion and Vulnerable Patients

Safety net populations include large proportions of patients with limited health literacy (LHL), limited English proficiency (LEP), and polypharmacy—all risk factors for misunderstanding the instructions or “sig” on prescription drug labels [53–56]. This presents particular risks with high alert medications such as insulin, where the risk of severe ADE (including hypoglycemia and death) with improper timing of insulin administration is much greater than for most medications. However, the risk of sig confusion also extends to other commonly prescribed oral hypoglycemic, antihypertensive, and lipid-lowering medications that are required for cardiometabolic control in diabetes. In examining a common diabetes “sig” instruction “take two tablets by mouth twice daily,” only 36% of patients could correctly demonstrate this instruction, with higher odds of incorrect demonstration among those with limited health literacy [53]. In addition, patients with limited English proficiency have significantly increased odds of reporting difficulty in understanding prescription drug labels [56].

EHRs offer an important opportunity to increase prescribing safety by eliminating these confusing sigs. Universal medication schedule (UMS) is a “plain-language” approach to standardizing and simplifying medication instructions to support safe and effective prescription drug use, highlighted by the Institute of Medicine as best practice for caring for patients across the health literacy spectrum [57]. All patients—particularly those with LHL and LEP—are more likely to interpret accurately and demonstrate comprehension of UMS instructions compared with current standard instructions [58–60]. A recent trial of patient-centered UMS prescription labels showed that LHL patients had higher odds of adhering to medications, compared with those receiving standard labels [60].

Unfortunately, despite strong support for the use of UMS labeling from stakeholders such as the National Council on Prescription Drug Programs (NCPDP) and the National Association of Boards of Pharmacy [61], EHR vendors have not incorporated UMS as their standard instruction format, instead prepopulating medication instructions using more confusing older jargon [62]. Moreover, for more complex medication instructions, such as regimens involving insulin, the default limit of 140 characters of the direction field presents a barrier to prescribers or health systems adding their own UMS instructions.

In the case of Mrs. D, the use of UMS language would have prevented Mrs. D from using a mealtime insulin at bedtime. Ideally, Dr. P should have been offered a menu of common UMS instructions when she prescribed Novolin R insulin.

Given the wealth of evidence suggesting that UMS can increase comprehension, medication safety, and adherence for safety net patients [57–60], combined with the clear direction in which labeling requirements are headed, electronic prescription vendors and EHRs should convert their standardized drug frequencies to universal medication schedule (UMS) language.

Finally, language-concordant prescriptions in several different languages have been shown to result in increased comprehension of instructions by patients [63, 64]. EHR vendors and policy makers should partner with implementation researchers and innovative health information technology platforms to incorporate language-concordant instructions for limited English proficiency patients [63–65].

### 3.4. Missed Opportunity to Facilitate Clinically Meaningful Collaboration with Pharmacists

Community pharmacists have a unique perspective on electronic prescribing due to their significant responsibilities with the downstream result of the electronic prescriptions [17, 66]. Pharmacists are tasked with clarifying the often conflicting instructions within the prescription, and successful implementation of EHRs can improve both the quality of electronic prescriptions and the quality of communication with pharmacists facilitated by EHRs [66].

First, as described above, EHR electronic prescribing vendors should adopt RxNorm to facilitate best practices implementation of both generic and brand names within the EHR and the medication label, to minimize confusion and error when switching formulations is required [12, 45, 46].

Second, EHRs and electronic prescribing vendors should focus on enhancing bidirectional communication between prescribers and pharmacists, integrating pharmacists electronically into the healthcare team supporting patients [16]. Providers currently use free-text electronic prescribing fields to communicate a diverse set of information to pharmacists, including requests to discontinue refills because the per-message feedisincentivizes pharmacies from accepting structured discontinuation messages [33–35]. Policies to remove this financial barrier will improve safety due to adverse drug events from failed discontinuation and allow providers to focus on conveying clinically meaningful information not captured through structured fields. Researchers have also uncovered several other categories of communication from prescribers to pharmacies, using text mining to codify the diverse wording of free-text prescriptions instructions [67]. Further research is needed about how to codify and convey these instructions through optimal design of EHR computer order entry and EHR-pharmacy communication interfaces to reduce omission and dosing errors and to help pharmacists discover potential prescription errors.

In addition, despite pharmacists' role in clarifying potentially erroneous prescriptions, pharmacists still lack convenient and efficient ways to reach prescribing providers. Pharmacists play significant roles uncovering barriers to medication safety and adherence for diverse and underserved LHL populations, through personalized education, and phone outreach [68–72]. Pharmacists and pharmacy technicians need platforms beyond faxing to convey this valuable information and the pharmacist team's interventions to prescriber care teams, thus expanding the patient-centered medical home into the patient's neighborhood. While policy leaders have lamented EHRs' overall failure to acknowledge the important contribution of the entire patient care team in providing care [73], improved EHR-pharmacy integration should also facilitate collaboration with the pharmacy teams who work closely with patients in their communities [17].

## 4. Conclusion

EHRs have yet to realize their promise to improve the quality, safety, and patient-centeredness of diabetes medication prescribing. In fact, the literature has prompted enough concern that informatics experts are calling for changes in EHR platforms, including better provider-centered design and usability testing of prescribing fields, tools for minimizing internal prescription discrepancies or omitted data, and enhancing provider training on the optimal use of EHRs [12, 16, 74]. The Office of the National Coordinator for Health Information Technology (ONC) is leading efforts to bring together EHR vendors, informatics leaders, and healthcare systems towards improving medication safety [75].

To improve the medication safety and health of safety net patients with diabetes—including LHL and LEP populations—we recommend policy changes to facilitate the following: (1) the adoption of RxNorm to reduce patient and prescriber confusion about medication names; (2) focused stakeholder engagement to standardize the naming and picklist options for high alert medications such as insulin; (3) the widespread implementation of the universal medication schedule and language-concordant labels, with the expansion of electronic prescription 140-character limit, to improve patient comprehension of how and when to take their medications; and (4) enhanced bidirectional communication with pharmacy partners, enabling pharmacies to receive “discontinuation” messages without cost and improving interoperability to allow pharmacist communication back to providers. Finally, informatics and implementation researchers should include safety net healthcare systems in examining the specific positive and negative consequences of EHR tools such as electronic prescribing in the care of diverse vulnerable populations.

*EHR-Facilitated Support of Mrs. D*

*Mrs. D, a 67-year-old Latina woman, suffers from thirst and frequent urination, with a hemoglobin A1c that jumped from 7.5% to 9.7%. Her community pharmacist sends a message to Dr. P stating that Mrs. D was asking her questions about pioglitazone side effects, reporting swollen face and legs. So, Dr. P brought Mrs. D in for a visit and counseled Mrs. D about adding a short-acting mealtime insulin to her metformin and glargine regimen. Dr. P searches the EHR RxNorm database for regular insulin and chooses a version covered by Mrs. D's insurance, but indicating flexibility for pharmacists to fill the least expensive formulation of regular insulin. After choosing the medication, the default instructions read “Take — units with breakfast and take — units with dinner,” allowing Dr. P to fill in the correct number of units for each dose. Dr. P also selects a “STOP” message for pioglitazone, which also allows her to record this adverse reaction automatically in Mrs. D's charts in both Dr. P's EHR and the pharmacy's electronic system. Mrs. D's after-visit summary includes an easy-to-read Spanish version of her medication schedule, with her medications automatically sorted by therapeutic indication. Similarly, the community pharmacist technician knows Mrs. D prefers instructions in Spanish and prints the prescription label in Spanish with the UMS instructions. Mrs. D is able to teach back both to Dr. P and to the community pharmacist how she will stop her old medication and how she will take her new medication, expressing thanks for the support from her entire care team.*
